# Supplementary figures and images for: Metabolite identification, tissue distribution, excretion and preclinical pharmacokinetic studies of ET-26-HCl, a new analogue of etomidate
Source: R Soc Open Sci. 2020 Feb 12;7(2):191666. doi: 10.1098/rsos.191666 (PMC7062083; doi:10.1098/rsos.191666)

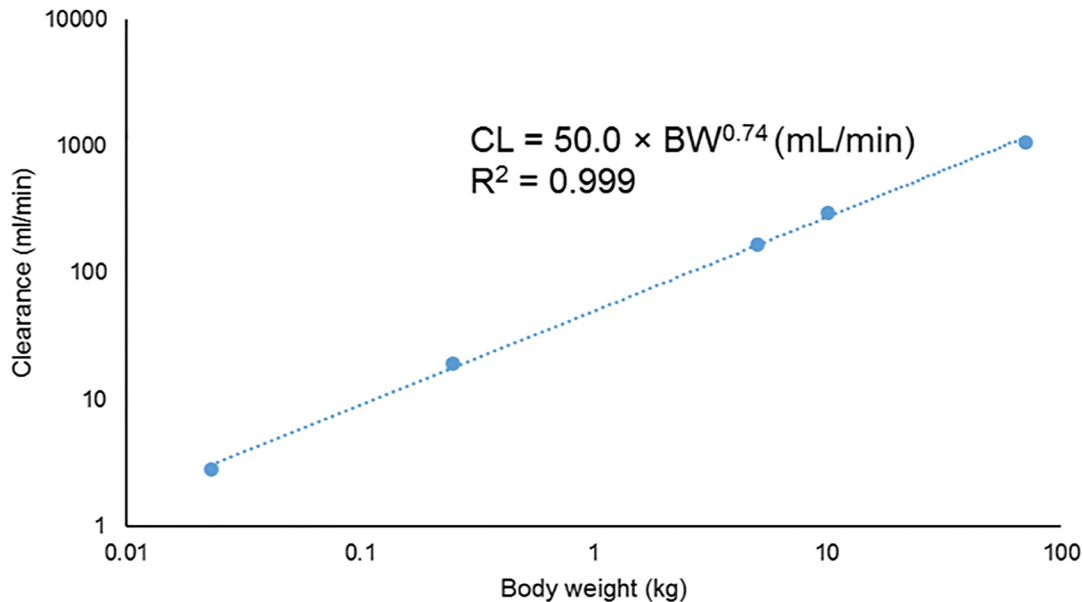

Supplement: Supplementary figures from “Metabolite Identification, Tissue Distribution, Excretion and Preclinical Pharmacokinetic Studies of ET-26-HCl, a New Analog of Etomidate” [file rsos191666supp1.zip › 20200110-supplementary figures-PDF/Fig.S3-pdf]

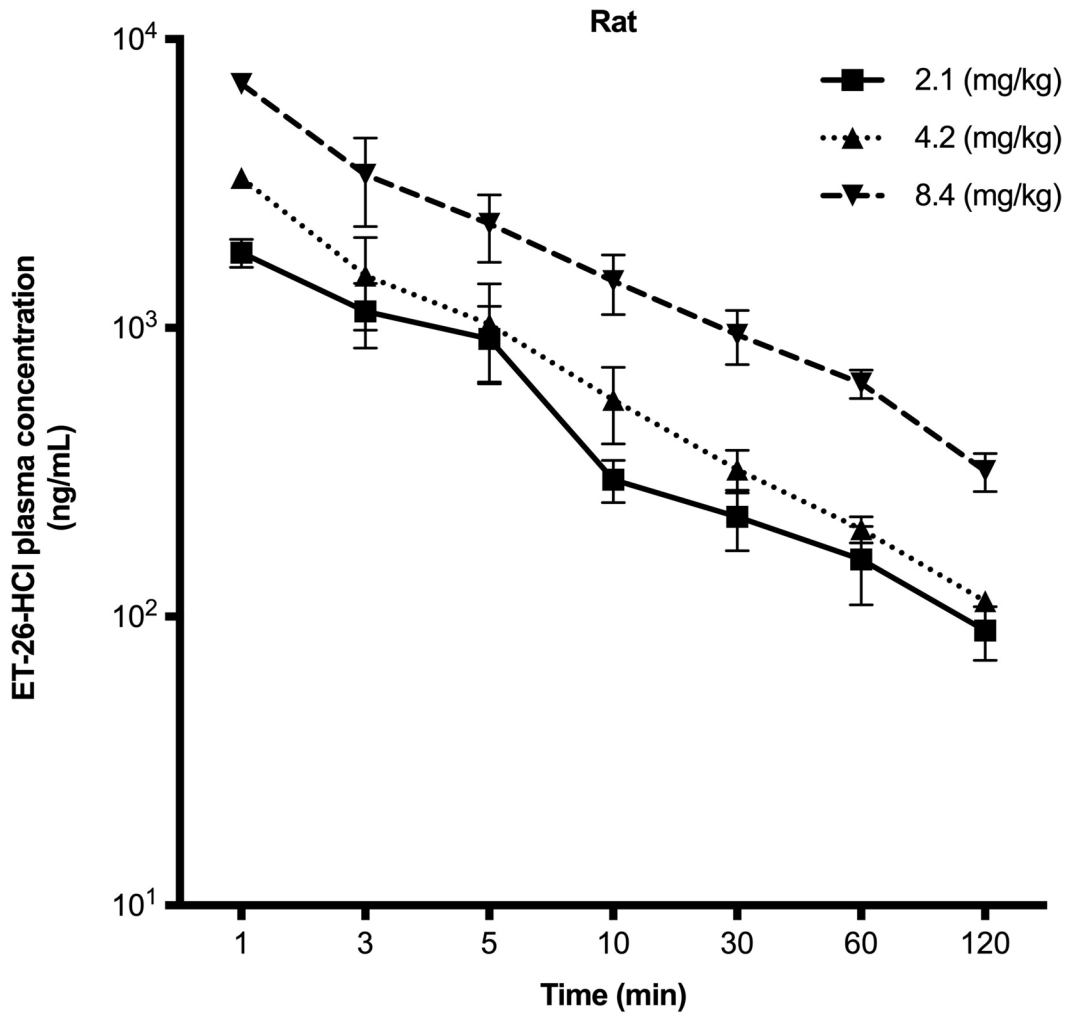

Supplement: Supplementary figures from “Metabolite Identification, Tissue Distribution, Excretion and Preclinical Pharmacokinetic Studies of ET-26-HCl, a New Analog of Etomidate” [file rsos191666supp1.zip › 20200110-supplementary figures-PDF/Fig.S2-pdf]

ET-26-HCL

ET-26-acid

Gabapentin

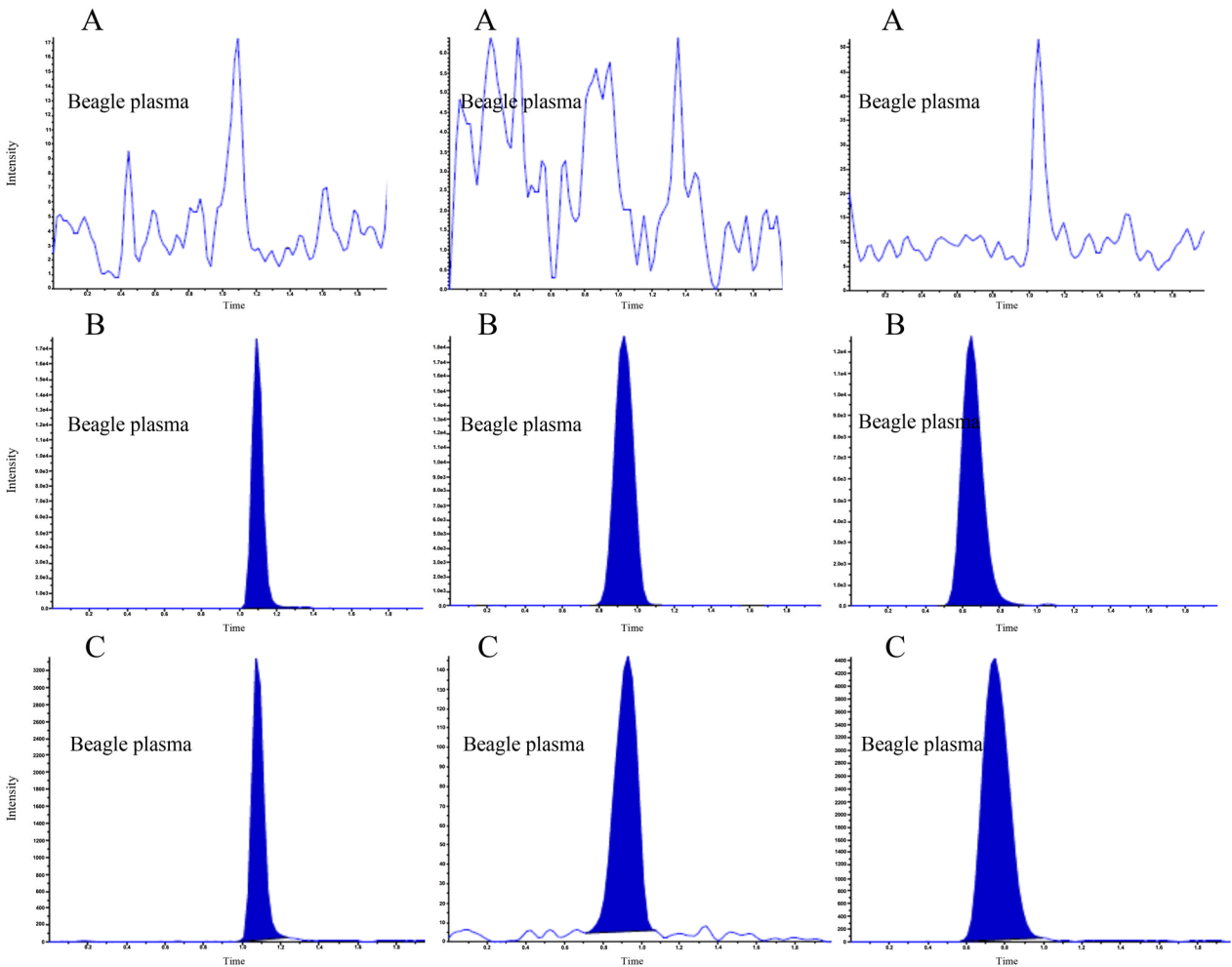

ET-26-HCL

ET-26-acid

Gabapentin

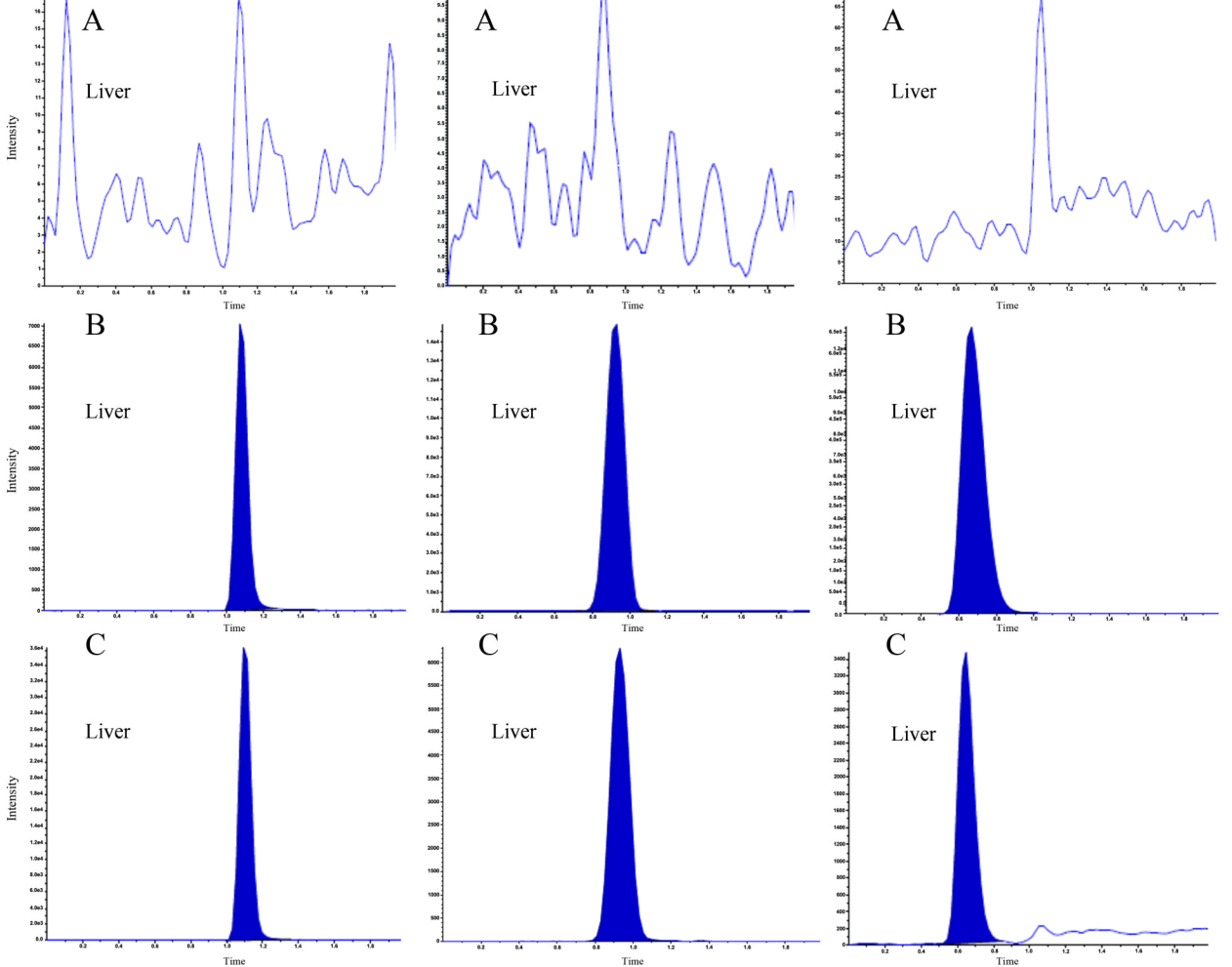

ET-26-HCL

ET-26-acid

Gabapentin

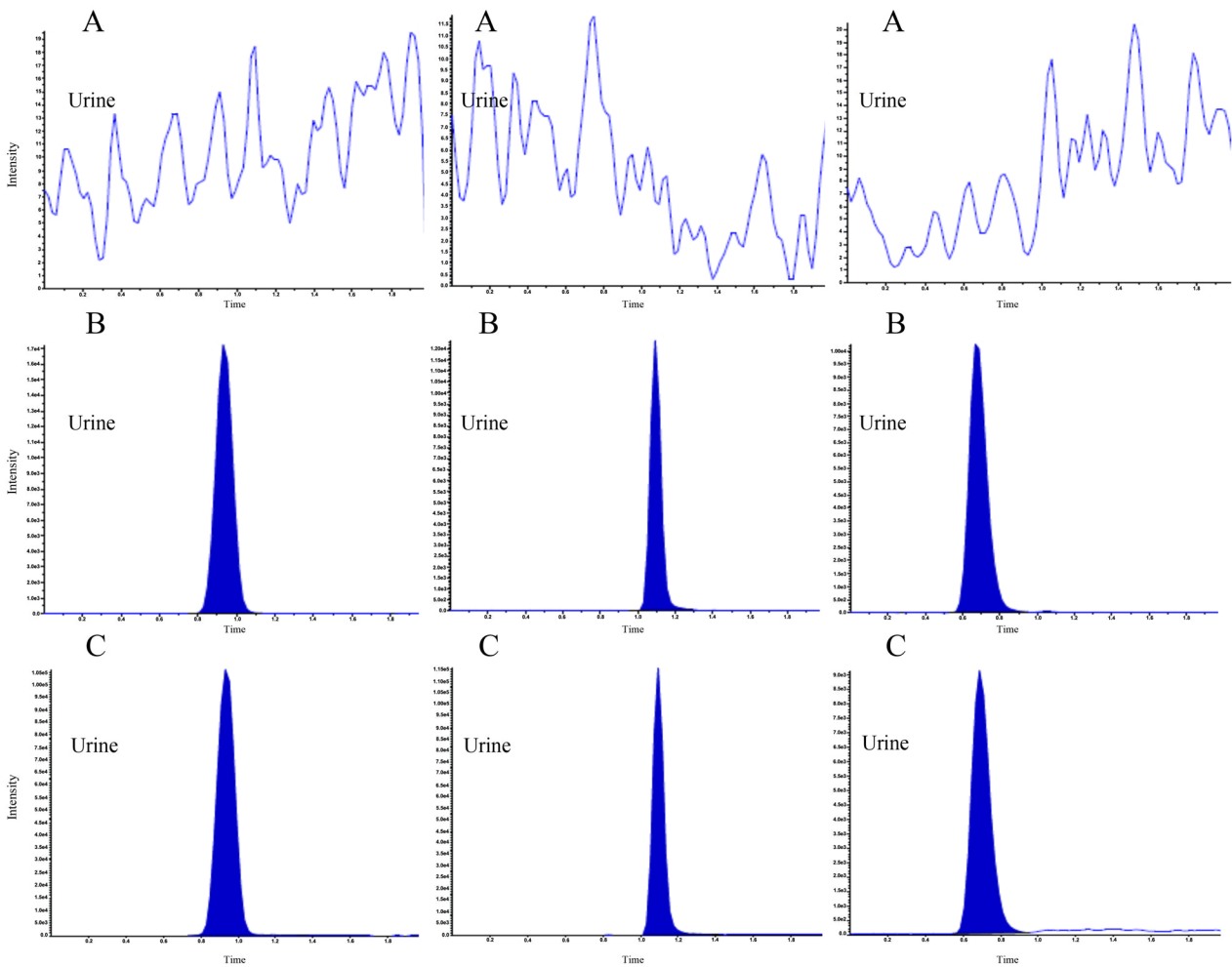

Supplement: Supplementary figures from “Metabolite Identification, Tissue Distribution, Excretion and Preclinical Pharmacokinetic Studies of ET-26-HCl, a New Analog of Etomidate” [file rsos191666supp1.zip › 20200110-supplementary figures-PDF/Fig.S1-pdf]
