# Supplementary material for: Metabolite identification, tissue distribution, excretion and preclinical pharmacokinetic studies of ET-26-HCl, a new analogue of etomidate
Source: R Soc Open Sci. 2020 Feb 12;7(2):191666. doi: 10.1098/rsos.191666 (PMC7062083; doi:10.1098/rsos.191666)
Supplement: Supplementary tables from "Metabolite Identification, Tissue Distribution, Excretion and Preclinical Pharmacokinetic Studies of ET-26-HCl, a New Analog of Etomidate” [file rsos191666supp2.docx]

| Matrix | Compound | Concentration (ng/ml) | Measured concentration (ng/ml) | | Accuracy (%) | Extraction recovery  (%) |
| --- | --- | --- | --- | --- | --- | --- |
|  |  |  | Intra-day (n=6) | Inter-day (n=18) |  |  |
| Plasma  (Beagles) | ET-26-HCL | 44.9 | 42.6±3.4 | 41.7±3.6 | 94.8±7.5 | 70.9±7.9 |
|  |  | 2246 | 2042±125.9 | 2103.3±214.3 | 90.9±5.6 | 85.5±4.3 |
|  |  | 3743 | 3706±296.1 | 3503.6±256.1 | 99.0±7.9 | 95.1±4.6 |
|  | ET-26-acid | 38.2 | 36.9±4.2 | 36.5±2.7 | 96.4±10.8 | 79.8±8.8 |
|  |  | 1912 | 1872±71.9 | 1845.9±74.6 | 106.8±6.6 | 90.5±2.6 |
|  |  | 3186 | 2844±149.1 | 2853.3±120.8 | 89.3±4.7 | 96.6±6.1 |
| Liver (Rats) | ET-26-HCL | 39.1 | 39.9±3.0 | 40.0±3.8 | 108.3±9.2 | 79.5±2.8 |
|  |  | 1957 | 1982±76.5 | 1852.1±144.8 | 101.3±3.9 | 92.1±8.5 |
|  |  | 3262 | 2967±183.2 | 2950.6±151.1 | 91.0±5.5 | 96.2±7.8 |
|  | ET-26-acid | 35.8 | 36.7±3.9 | 38.1±3.1 | 102.5±10.8 | 100.0±14.3 |
|  |  | 1789 | 1713±168.9 | 1863.8±152.1 | 108.4±5.2 | 96.3±3.6 |
|  |  | 2982 | 2845±70.25 | 3052.6±186.9 | 95.4±2.4 | 92.9±3.0 |
| Urine (Rats) | ET-26-HCL | 104.5 | 102.6±4.1 | 101.7±6.3 | 100.2±6.2 | 103.8±13.4 |
|  |  | 4181 | 4183±141.9 | 4174±118.2 | 90.0±3.8 | 90.6±5.8 |
|  |  | 6968 | 7305±275.9 | 7107±191.4 | 96.4±6.5 | 103.4±2.5 |
|  | ET-26-acid | 74.7 | 80.4±2.1 | 78.8±4.9 | 107.5±2.9 | 99.7±8.3 |
|  |  | 3737 | 3822±109.8 | 3711±99.3 | 102.3±2.9 | 92.8±4.7 |
|  |  | 6228 | 5679±115.0 | 6101±130.6 | 91.2±1.9 | 105.4±4.7 |

**Table S1** Intra-day and inter-day accuracy, precision and extraction recovery for ET-26-HCL and ET-26-acid (n = 6)

**Note:** Data are presented as mean ± SD.

**table S2** The stability of ET-26-HCL and ET-26-acid in beagle plasma (n = 3)

| Compound | Conditions | Concentration  (ng/mL) | Measured concentration  (Mean ± SD) | RSD  (%) |
| --- | --- | --- | --- | --- |
| ET-26-HCL | 4◦C for 3 h | 44.92 | 48.79±3.275 | 6.71 |
|  |  | 3743 | 3977±294.1 | 7.40 |
|  | Room temperature 3 h | 44.92 | 47.91±4.952 | 10.34 |
|  |  | 3743 | 3599±136.1 | 3.78 |
|  | –80 ◦C for 30 days | 44.92 | 44.67±4.795 | 10.73 |
|  |  | 3743 | 3989±229.1 | 5.74 |
|  | Freeze and Thaw Cycle (3) | 44.92 | 47.75±3.427 | 7.18 |
|  |  | 3743 | 3722±240.3 | 6.45 |
|  | | | | |
| ET-26-acid | 4◦C for 3 h | 38.24 | 39.92±2.904 | 7.28 |
|  |  | 3186 | 2890±174.8 | 6.05 |
|  | Room temperature 3 h | 38.24 | 38.29±4.088 | 10.68 |
|  |  | 3186 | 2890±117.6 | 4.07 |
|  | –80 ◦C for 30 days | 38.24 | 35.84±1.899 | 5.30 |
|  |  | 3186 | 3049±391.4 | 12.84 |
|  | Freeze and Thaw Cycle (3) | 38.24 | 36.23±2.568 | 7.09 |
|  |  | 3186 | 2770±157.7 | 5.69 |

**table S3** The linear range of this LC-MS/MS method for ET-26-HCl and for ET-26-acid

| Compound | Species | Sample | The linear range (ng/mL) |
| --- | --- | --- | --- |
| ET-26-HCL | Dog | Plasma | 21.82~4346 |
|  | Rat | Heart | 21.76~4352 |
|  |  | Spleen |  |
|  |  | Lung |  |
|  |  | Intestine |  |
|  |  | Colon |  |
|  |  | Kidney |  |
|  |  | Brain |  |
|  |  | Fat |  |
|  |  | Stomach |  |
|  |  | Liver |  |
|  |  | Muscle |  |
|  |  | Plasma |  |
|  |  | Testis |  |
|  |  | Ovary |  |
|  |  | Urine | 54.40~8704 |
|  |  | Bile |  |
|  |  | Feces | 13.60~1088 |
| ET-26-acid | Dog | Plasma | 18.56~3712 |
|  | Rat | Heart | 21.62~4324 |
|  |  | Spleen |  |
|  |  | Lung |  |
|  |  | Intestine |  |
|  |  | Colon |  |
|  |  | Kidney |  |
|  |  | Brain |  |
|  |  | Fat |  |
|  |  | Stomach |  |
|  |  | Liver |  |
|  |  | Muscle |  |
|  |  | Plasma |  |
|  |  | Testis |  |
|  |  | Ovary |  |
|  |  | Urine | 46.35~7416 |
|  |  | Bile |  |
|  |  | Feces | 11.59~927 |

**Table S4** Main pharmacokinetic parameters of ET-26-HCl after intravenous administration of ET-26-HCl to rats

| Parameters | Dose (mg/kg) | | |
| --- | --- | --- | --- |
|  | 2.1 | 4.2 | 8.4 |
| λ (1/h) | 0.66 ± 0.24 | 1.20 ± 0.36 | 0.78 ± 0.12 |
| t_1/2_ (h) | 1.09 ± 0.21 | 0.66 ± 0.17 | 0.85 ± 0.14 |
| C_max_ (mg/L) | 1.82±0.20 | 3.31±0.21 | 7.01±0.36 |
| AUC_0-t_ (mg/L*min) | 28.47 ± 3.41 | 41.70 ± 3.61 | 110.92 ± 9.04 |
| Vd (L/kg) | 5.37±0.72 | 5.24±1.33 | 4.67±0.99 |
| CL (L/h/kg) | 3.48 ± 0.66 | 5.52 ± 0.48 | 3.84 ± 0.3 |

**Note:** Data are presented as mean ± SD, n=6 for rats. Data of rats was cited from our previous study [11].

**Abbreviations:** AUC_0-t_, area under the plasma concentration-time curves from zero to the last measurable point; CL, clearance; C_max_, maximum concentration; t_1/2_, half-life; Vd, volume of distribution.
